# Book Reviews

**Published:** 1910-04

**Authors:** 


### BOOK REVIEWS

A PRACTICAL TREATISE ON FRACTURES AND DISLOCATIONS. By Lewis A. Stemson, B. A., M. D., L. L. D. (Yalen). Prof. Surgery Cornell University Medical College, N. Y.; Surgeon the New York and Hudson Street Hospitals, &c., &c. Sixth edition revised and enlarged, with 361 illustrations and 65 plates in monotint. Pp. 876. Lea & Febiger, Publishers, Philadelphia.

This now classical work on fractures and dislocations has needed little revision in the present volume, the additions to which largely represent the results of the injuries of the small bones of the carpus and tassus and include a new subhead, the mid-caspal fracture-dislocation, the recognition of which has come largely through investigations aided by X-ray examinations.

Certain important additions relative to treatment have also been made.

The entire subject is discussed in such a thorough and masterly manner little can be said in the way of criticism. The many excellent cuts add greatly to the clearness of the text.

It is a splendid book and one which can be given unstinting praise.

---

ANIMAL EXPERIMENTATION. "To the Laborers in the Fields of Biologic Sciences, Who Wrest from Nature Her Secrets, to the End that Life shall be more Sweet, that Pain shall be relieved, that Childhood, Youth, and Age shall be Absolved from the Hazards of Disease, that Death shall be Postponed, and the Light of Truth shall Fall upon the Dark places."

With the above paragraph as dedication lines, James Peter Warbosse has presented to the public a book entitled "The Conquest of Disease Through Animal Experimentation"* which would suppress antivivisection if the followers of this misguided sect would but carefully read and consider its teachings. "There is no knowledge so useful to man as knowledge of himself . . . . as life is the loftiest consumation of natural energy," human life should be the supreme object of human interest. And yet as John Dewey has said, scientific enquiry has been the chief instrumentality in bringing man from barbarism to civilization, from darkness to light; while it has incurred, at every step, determined opposition from the powers of ignorance, misunderstanding and jealousy. Who of us can enter a home where a devoted mother or wife or a dear little child lies stricken and think for a moment of the rabbits or guinea pigs that have been subjected to scientific experimentation in order that the most useful remedies might be garnered into our all too meager storehouse of curative treatment? Do not even the rabid, poorly balanced antivivisectionists gladly welcome for their children or relatives the acurate methods we have learned through experimentation? Are they not more to be "pited than censured" for their narrow little view of life and for their gross inconsistency in eating game and animal flesh and complacently allow millions of animals to be slain in slaughter houses without so much as protest? Can they really have any grasp of the situation when they continue their perineal howl about the few small animals which are painlessly or almost painlessly used in the comparatively few scientific laboratories where such work is done?

To even a physician the vast assistance animal experimentation has been to scientific medicine is not realized until after perusing a systematic recital of the real advances and the manner in which they were made. Physicians should inform themselves upon these points in order to assist in arousing in their patients a realization of the assistance animal experiemntation is to medicine and to show the sophistry and falsehood in the reasoning of the anti-vivisectionists. Thus can public sentiment be trained in sound facts so that it will not be mislead by well meaning but little-minded cranks and obstructionists.

*D. Appleton & Co., New York.

---

THE DISEASES OF INFANCY AND CHILDHOOD. Designed for the use of Students and Practitioners of Medicine. By Henry Koplik, M. D., attending physician to Mount Sinai Hospital; consulting physician to the Hospital for Deformatories, &c., &c. Third edition revised and enlarged. Illustrated with 204 engravings and 39 plates in color and monochrome. Pp. 944. Lea & Febiger, Publishers, Philadelphia.

An introduction to this book to the medical profession is unnecessary, as it is already well and favorably known. During the past three years since the previous edition appeared many advances have been made in pedeatrics and have cleared up many of the obscure problems. These have been added to the volume under review with, of course, a full description of the indications and technic of administering of lexners serum for the treatment of meningitis. In the treatment and diagnosis of infectious diseases there is much that is new and yet well tried in practice. Infant feeding, which has advanced as to our understanding of certain disturbances of nutrition, has been carefully revised. Many new illustrations are found in this edition. The work of the publishers is well up to their usual high standard and the book as a whole may be highly commended.

